# Imaging the neural mechanisms of TMS neglect-like bias in healthy volunteers with the interleaved TMS/fMRI technique: preliminary evidence

**DOI:** 10.3389/fnhum.2012.00326

**Published:** 2012-12-17

**Authors:** Raffaella Ricci, Adriana Salatino, Xingbao Li, Agnes P. Funk, Sarah L. Logan, Qiwen Mu, Kevin A. Johnson, Daryl E. Bohning, Mark S. George

**Affiliations:** ^1^Brain Stimulation Laboratory, Department of Psychiatry and Behavioral Sciences, Medical University of South CarolinaCharleston, SC, USA; ^2^Department of Psychology and Neuroscience Institute of Turin, University of TurinTurin, Italy; ^3^The Center for Advanced Imaging Research, Medical University of South CarolinaCharleston, SC, USA; ^4^Stanford Systems Neuroscience and Pain Laboratory, Stanford School of MedicinePalo Alto, CA, USA

**Keywords:** TMS, fMRI, spatial attention, neglect, visuospatial orienting

## Abstract

Applying a precisely timed pulse of transcranial magnetic stimulation (TMS) over the right posterior parietal cortex (PPC) can produce temporary visuo-spatial neglect-like effects. Although the TMS is applied over PPC, it is not clear what other brain regions are involved. We applied TMS within a functional magnetic resonance imaging (fMRI) scanner to investigate brain activity during TMS induction of neglect-like bias in three healthy volunteers, while they performed a line bisection judgment task (i.e., the landmark task). Single-pulse TMS at 115% of motor threshold was applied 150 ms after the visual stimulus onset. Participants completed two different TMS/fMRI sessions while performing this task: one session while single-pulse TMS was intermittently and time-locked applied to the right PPC and a control session with TMS positioned over the vertex. Perceptual rightward bias was observed when TMS was delivered over the right PPC. During neglect-like behavior, the fMRI maps showed decreased neural activity within parieto-frontal areas, which are often lesioned or dysfunctional in patients with left neglect. Vertex TMS induced behavioral effects compatible with leftward response bias and increased BOLD signal in the left caudate (a site which has been linked to response bias). These results are discussed in relation to recent findings on neural networks subserving attention in space.

## Introduction

In cognitive neuroscience, the non-invasive technique of transcranial magnetic stimulation (TMS) can be used in healthy participants to temporarily disrupt the activity of a focal brain region and test its function (Pascual-Leone et al., 2000). It is assumed that the effects of TMS causally suggest the involvement of the stimulated cortex in the execution of the observed behavior and can reveal whether a given region is necessary for the occurrence of a motor, perceptual, or cognitive event. This use of TMS can partially overcome the limitations posited by correlating lesion locations to neuropsychological symptoms in neurological patients (i.e., the large extension of natural lesions, the remote effects of the diaschisis, and brain reorganization). However, the neural correlates of TMS virtual neurology (Rafal, 2001) are still poorly understood.

Over the past decade, several authors have investigated the neural basis of spatial attention in healthy volunteers by using TMS to reproduce subtle and transitory biases mimicking symptoms of visuo-spatial neglect, a neuropsychological disorder of contralesional spatial attention and representation (Bisiach and Berti, 1995), which often follows right parietal brain lesions (Vallar and Perani, 1986; Marshall et al., 2002). However, none of these studies have provided direct evidence of brain activity changes underlying TMS behavioral effects. Here, we used the interleaved TMS/fMRI technique in healthy volunteers to directly investigate TMS effects on brain activation during induction of neglect-like bias.

In healthy participants, neglect-like behavior has been induced by applying TMS over the right posterior parietal cortex (Fierro et al., 2000, 2001, 2006; Brighina et al., 2002; Ellison et al., 2004; Bjoertomt et al., 2009; see Sack, 2010 for a review). In most of these studies, visuo-spatial performance has been measured on a line bisection judgment task (i.e., the landmark task), originally employed in patients with neglect (Milner et al., 1993; Bisiach et al., 1998). The induction of neglect-like attentional biases on the landmark task by right posterior parietal (PPC) TMS, has been shown to be side (Fierro et al., 2000) and site (Ellison et al., 2004) specific, and not to depend on indirect sensory effects, such as right lateralized noise or scalp-tapping sensation, which could potentially act as exogenous cues. Moreover, right PPC TMS effects on visuo-spatial attention are time specific. Fierro et al. (2001) applied single-pulse TMS at different time intervals (150 ms, 225 ms, and 300 ms) over the right PPC and the right prefrontal cortex to obtain information on the timing of activity in these regions during visuo-spatial processing. TMS delivered over the right PPC 150 ms after the visual stimulus onset induced transitory rightward neglect, while right frontal TMS at these intervals did not affect the participants' behavior.

In this preliminary study, we used a single-pulse TMS protocol very similar to the one used by Fierro et al. (2001) in three healthy participants, to test the feasibility of applying TMS within an MRI scanner to actually measure the patterns of brain activity that occur during induction of a visuo-spatial bias. TMS was applied 150 ms after stimulus presentation over the right PPC and, in a control condition, over the vertex. Even though single-pulse TMS is less powerful than repetitive TMS (rTMS), it may provide cleaner information on brain activity changes concomitant to behavioral effects.

Concurrent TMS/fMRI studies have shown that TMS does not only affect the cortex underneath the coil, but also remote cortical and subcortical regions that are anatomically and/or functionally interconnected to the stimulated area (Bohning et al., 2000; Bestmann et al., 2003, 2004; see also Bestmann et al., 2008 and Ruff et al., 2009b). Recently, researchers have used concurrent TMS/fMRI to directly investigate causal interactions between fronto-parietal regions and the visual cortex underlying visuo-spatial attention (Ruff et al., 2006, 2008, 2009a; Blankenburg et al., 2010; Heinen et al., 2011). These studies demonstrated high spatial specificity for the effects of frontal and parietal TMS on the visual retinotopic cortex and right hemisphere predominance for the parietal site. Additionally, they showed that right PPC TMS effects were modulated by the visual stimulation and the current state of attention (Blankenburg et al., 2010; Heinen et al., 2011). In line with findings in humans, the feline literature has provided evidence that visuo-parietal online rTMS decreases metabolic activity (as measured through glucose uptake) on the site of stimulation and cortical and subcortical regions known to receive efferent projections from the stimulated cortex (Valero-Cabre et al., 2005, 2007).

To our knowledge, only two studies (Sack et al., 2007; Heinen et al., 2011) have used TMS/fMRI to image brain activity changes during performance of a visuo-spatial task in healthy participants. In Sack et al.'s study (Sack et al., 2007), increased Reaction Times on visuo-spatial angle judgments by short bursts of TMS over the right superior parietal lobule (SPL) were concomitant to decreased neural activity at the site of stimulation and in other near and remote interconnected right hemisphere regions [such as post-central gyrus and middle frontal gyrus, (MFG)]. In Heinen and colleagues' study (Heinen et al., 2011) TMS of the right angular gyrus (AG) facilitated reorienting to invalidly cued right visual targets, during an exogenously cued visuo-spatial attention task, and enhanced BOLD signal in the left AG and left retinotopic cortex.

In this initial study we used the interleaved TMS/fMRI technique in three healthy volunteers to directly investigate the specific impact of right PPC single-pulse TMS on brain activity changes during induction of neglect-like attentional rightward bias. We used a version of the landmark task, which has been previously employed in neglect patients (Bisiach et al., 1998, 1999) and healthy participants (Brighina et al., 2002) to disentangle perceptual and response biases underlying line bisection performance. Given the small sample size, due to the difficulty in combining TMS with fMRI recording during performance of a behavioral task, individual analyses are also presented and considered beside group analyses. Indeed, with small sample sizes, group results might be largely affected by individual findings.

Results from this pilot study may lay the groundwork for follow up investigations to determine brain activation changes occurring during induction of TMS behavioral effects in relation to other visuo-spatial tasks or brain areas thought to be involved in the neural circuitry of spatial attention and representation.

## Methods

### Participants

Participants were three right-handed healthy women with a mean age of 32 years. They had normal vision and no history of neurological or psychiatric illness. All were screened for MRI and TMS compatibility. Participants were given a detailed explanation of the procedure and signed a written informed consent form approved by the Medical University of South Carolina Institutional Review Board.

### Apparatus and stimuli

**Figure 1:**
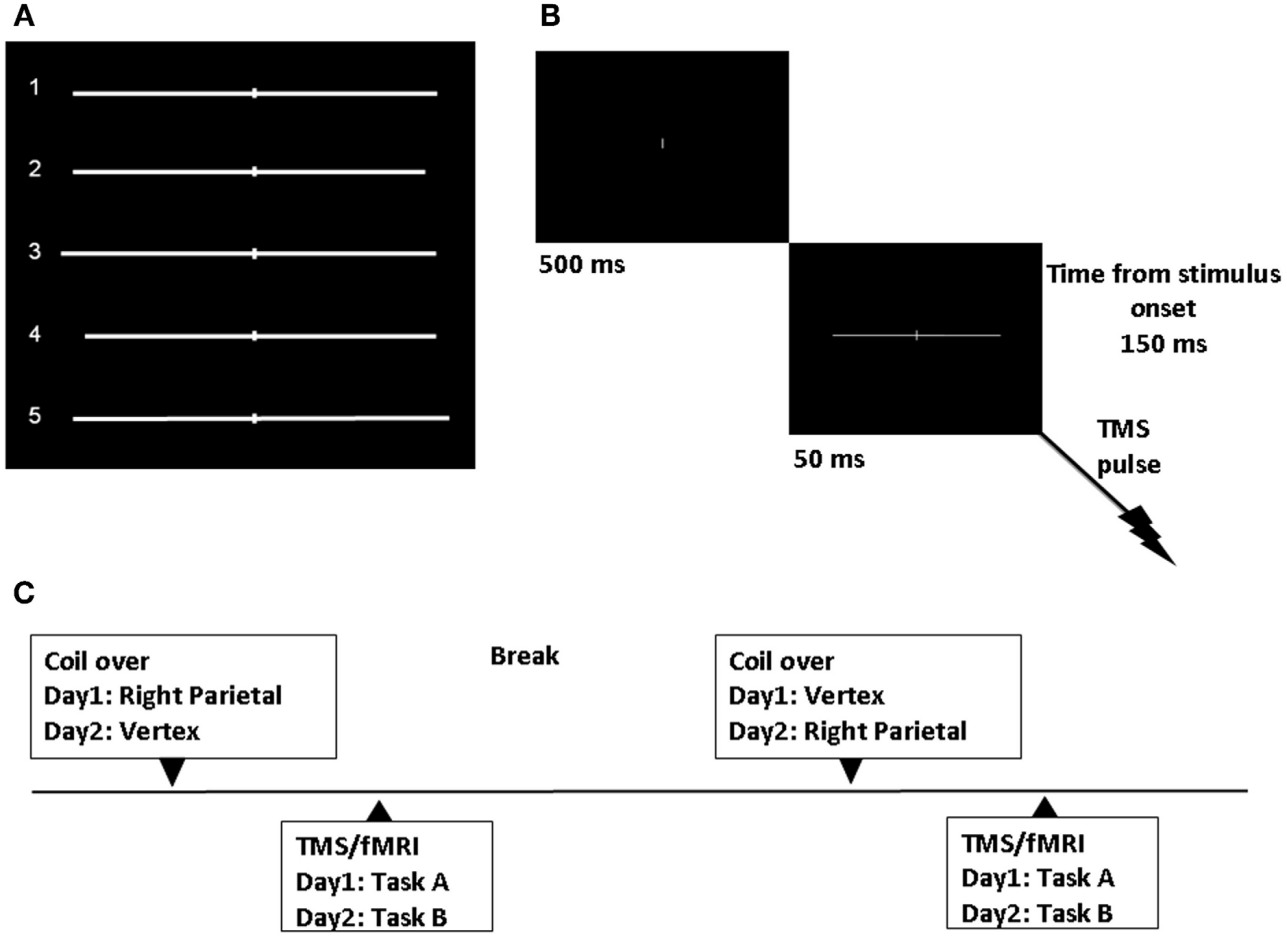
**Experimental design. (A)** Symmetrically and asymmetrically bisected lines used in the study. Line 1 (symmetrically bisected): left segment 10.13° of visual angle/right segment 10.13°; Line 2 (left-elongated): left segment 10.13°/right segment 9.47°; Line 3 (left-elongated): left segment 10.79°/right segment 10.13°; Line 4 (right-elongated): left segment 9.47°/right segment 10.13°; Line 5 (right-elongated): left segment 10.13°/right segment 10.79°. **(B)** Sequence of events. TMS single-pulse was delivered 150 ms after visual stimulus onset. **(C)** Time course of the experiment.

Stimuli were generated through the Eprime software (Psychology Software Tools, Inc., Sharpsburg, PA) and back-projected onto a screen at the feet of the magnetic bore by a LCD projector. Participants viewed the stimuli through a mirror attached to the head coil. Stimuli consisted of a white horizontal line with 0.14° of visual angle thickness and approximately 20° of visual angle length transected by a 0.14° thick and 1.37° high vertical bar and presented on a black background. Six lines were symmetrically bisected with both the left and right segment of 10.13° of visual angle. Six lines were asymmetrically bisected to the right of the true center (3 lines with a left segment of 10.13° and a right segment of 9.47° of visual angle; 3 lines with a left segment of 10.79° and a right segment of 10.13° of visual angle). Six lines were asymmetrically bisected to the left of the true center (3 lines with a left segment of 9.47° and a right segment of 10.13°; 3 lines with a left segment of 10.13° and a right segment of 10.79° of visual angle). See Figure 1A.

### Procedure

We applied single-pulse TMS and obtained real-time fMRI scans while participants performed a line bisection judgment task (the landmark task). Single-pulse TMS at 115% of resting motor threshold (RMT), was delivered 150 ms after the visual stimulus onset (Figure 1B). The participants' individual RMT (for the right abductor pollicis brevis) was determined with the Magstim stimulator while lying on the scanner bed. The RMT was defined as the lowest stimulus intensity to elicit a minimum of five muscle twitches from ten consecutive stimuli applied over the motor hot spot. For the three subjects the average RMT was 64.3 (SD = 9.29) of maximum machine output.

Each trial started with a white 0.14° of visual angle thick and 1.37° high vertical bar appearing on the center of a black screen. Participants were instructed to focus on this central vertical line. After 500 ms a pre-bisected horizontal line was presented for 50 ms. The vertical bar bisecting the line was presented at the center of the screen.

Participants were asked to estimate the length of the bisected line segments, according to opposite task instructions. That is, in task A, they had to report the segment that appeared to be subjectively longer, and in task B the segment that appeared to be subjectively shorter. Participants reported which of the two line segments was longer or shorter by pressing the left-sided or right-sided key of an MRI compatible glove using their index or middle finger (respectively) of the right hand. They were asked to respond as quickly as they could, but not to sacrifice accuracy for speed. We used two complementary tasks (longer or shorter segment) and only two possible responses (left or right segment) as originally used by Bisiach et al. (1998) in neglect patients rather than using the procedure by Fierro et al. (2001), in which a third response (equal) was allowed. The two complementary tasks were used to control whether TMS was biasing participants' performance at a perceptual (i.e., inducing consistent biases in the two opposite tasks) or a response level of spatial processing (i.e., inducing a tendency to consistently report one side of space, independent of task requests: for example, the segment ipsilateral to stimulation). Indeed, in neglect patients, deficits of spatial awareness can occur at input or output (i.e., “intentional neglect,” “directional hypokinesia” or response bias) stages of information processing (Coslett et al., 1990; Milner et al., 1993; Ricci and Chatterjee, 2004). As in Bisiach et al. (1998), only two possible responses were allowed even though some of the lines were symmetrically bisected. This was done to make the task more sensitive in detecting subtle changes in the participants' perception and the task more demanding.

Task A and B were performed on different days. For each task, participants completed two different TMS/fMRI conditions. In one condition, single-pulse TMS was intermittently and time locked applied to the right PPC. The PPC site was identified using anatomical skull landmarks (9 cm dorsal to the mastoid inion and 6 cm lateral) according to findings from a previous study using functional and anatomical procedures (Ellison et al., 2004). In Figure 1C the time course of the study is represented.

In order to provide *post-hoc* projections of the site of stimulation for each subject, MRI scans were co-registered with visible vitamin E on the subjects' head. *Post-hoc* projections showed that the PPC site overlaid the right AG in participant 1 (P1, approximate MNI coordinates: 51, −61, 51) and participant 3 (P3: 40, −75, 44), but it was located in a slightly higher cortical area, near the intra-parietal sulcus (IPS) in the SPL, in participant 2 (P2: 43, −61, 61). In the control condition, TMS was positioned over the vertex. As for previous studies (Li et al., 2010) the TMS coil was rigidly mounted in the MRI head coil with a specially designed TMS coil holder. Subjects wore plastic caps, on which their individual PPC and vertex spots were marked. Within each TMS/fMRI scan there were 36 trials. TMS was delivered during only half of the trials and it was “off” on the other half. “On” and “off” trials were given following one of two possible fixed pseudo-random orders. Coil location, Task, and stimuli orders were pseudo-randomized across subjects.

### Behavioral data analysis

Participants' reaction times for correct responses on the asymmetrically bisected lines and, separately, for responses on the symmetrically bisected lines were analyzed using the Wilcoxon test for dependent samples. For symmetrically bisected lines, reaction times of all responses were analyzed. The test was performed to compare the factors: TMS (on vs. off), stimulation condition (PPC vs. vertex), and task (A vs. B).

Participants' accuracy for the asymmetrically bisected lines and proportions of left vs. right choices for the symmetrically bisected lines were analyzed separately using the Fisher's exact test.

Participants' performance was also analysed according to the method proposed by Fierro et al. (2001). Participants' performance on each trial was scored as follows: 0 = correct responses on the asymmetrically bisected lines; 1 and 2 = rightward error due to left underevaluation (1 = right segment judged longer or left segments judged shorter on the symmetrically bisected line; 2 = right segment judged longer or left segment judged shorter on the rightward bisected line); −1 and −2 leftward error due to right underevaluation (−1 = right segment judged shorter or left segments judged longer on the symmetrically bisected line; −2 = right segment judged shorter or left segment judged longer on the leftward bisected line). Negative values indicated a leftward bias (right underevaluation) while positive values indicated a rightward bias (left underevaluation i.e., neglect-like bias). The non-parametric Sign Test was used to perform statistical analyses on these scores.

### Neuroimaging: data acquisition and data analysis

#### Combined TMS and fMRI

Combined TMS and fMRI acquisitions were performed in a Philips 3.0-T MRI scanner (Intera, Philips Medical System, The Netherlands) with an eight-channel SENSE head coil, using a standard gradient echo, echo planar imaging (EPI) fMRI sequence (flip angle = 90, TR/TE 2300/35 ms, FOV 230 mm, 23 3.5-mm-thick slices, 0.5-mm gap, matrix 64×64, Echospacing = 0.57 ms, Bandwidth = 2056). The fMRI time series consisted of 360 images preceded by six dummy images.

High-resolution anatomical images (Sense-head, sagittal, FOV 256 mm, RFOV(%) 100, Matrix scan 256, slice 180, slice thickness 1.00, TE = shortest, Flip angle 9.00, TR = shortest) were also acquired with fiducials (vitamin E capsule) placed over the right PPC. Participants wore earplugs for hearing protection and to reduce auditory responses.

TMS was applied using a MAGSTIM Super Rapid stimulator which generates biphasic electrical pulses of approximately 250-μ s duration. The pulses were delivered through a special non-ferromagnetic TMS coil of figure of eight design with an 8-m cable. The room setup was identical to *prior* TMS/fMRI studies from our group (Li et al., 2010). In particular, LabVIEW was running on a G4 computer to control the TMS timing, as well as visual stimuli presentation (Eprime software) through the integrated functional imaging system IFIS (Gainesville, Fla.). A CA- 210 Connector Accessory Enclosure from National Instruments was connected to the TMS and the G4 computer and served to precisely integrate the TMS pulses and visual presentation with the actual scan acquisition. TMS pulses and the fMRI sequence were interleaved as described before (Shastri et al., 1999). We employed a variable, jittered event-related design. The rest time between conditions varied from 20 to 25 s to minimize anticipation and to promote novelty. In other previous works (Shastri et al., 1999; Li et al., 2010), we observed artifacts in some slices when we initially tested the interleaved sequence. After several weeks of testing the sequence for this study, we were able to modify the exact timing of the TMS firing to be optimally placed between each slice uptake and overcome the problem of the artifacts.

#### fMRI data analysis

***Data preprocessing.*** MR scans were transferred into ANALYZE format with MRIcro (http://www.sph.sc.edu/comd/rorden/mircro.html) and then further processed in Matlab 7.5 (Mathworks, Sherborn, MA, USA) with SPM software 8 (The Wellcome Department of Cognitive Neurology, London, UK; http://www.fil.ion.ucl.ac.uk/spm). Default settings were used unless indicated otherwise. All volumes were realigned to the first volume. After realignment, the images were spatially normalized into a standard space with a resolution of 3-mm^3^ voxels using the averaged functional EPI image—the Montreal Neurological Institute (MNI) EPI template in SPM8. Subsequently, the data were smoothed with an isotropic 8-mm^3^ Gaussian kernel and high-pass filtered (cutoff period = 128 s).

***First-level analysis.*** At the individual subject level, the data were modeled with two conditions (TMS on, and TMS off), each modeled by a boxcar convolved with a synthetic hemodynamic response function. Contrasts were constructed to examine the two conditions vs. rest.

In order to analyze the effects of TMS separately for parietal and vertex stimulation at individual level, for each participant data were entered into a first level analysis (fixed effects analysis) with two repeated sessions. The first-level regression model consisted of a set of 4 regressors, 2 for TMS (on and off), and 2 for task (A and B), and convolved with the hemodynamic response function. Contrasts were constructed to examine TMS on vs. TMS off conditions. When not otherwise specified, the *t* maps were thresholded at *p* ≤ 0.001 (uncorrected), and cluster analyses were performed with a spatial extent threshold of 15 contiguous voxels (Friston et al., 1996).

***Group data analysis.*** Second-level analysis utilized the individual contrast images for simple effects from the first-level analysis. The differential effects of the experimental tasks were assessed with a three-way factorial design: TMS (on vs. off), site (PPC vs. vertex), and task (A vs. B). One sample *t*-test contrasts were constructed to examine the overall effect of site, task, and TMS and, for each stimulation condition (PPC and vertex), the effect of TMS during task execution. When not otherwise specified, the combined group *t* maps were thresholded at *p*≤ 0.001 uncorrected, cluster level *p* < 0.05 corrected, and cluster analyses were performed with a spatial extent threshold of 18 contiguous voxels.

## Results

### Behavior

On the asymmetrically bisected lines participants' RTs were significantly slower (Wilcoxon test *p* < 0.0001) during the vertex (680.14 ms SD = 139.87) with respect to the PPC condition (664.28 ms SD = 172.14) independently of on/off TMS trials, while no significant differences were observed for the other factors. RTs analyses for the symmetrically bisected lines showed similar results: participants' RTs were significantly slower (Wilcoxon test *p* = 0.004) for the vertex (768.07 ms SD = 148.75) with respect to the PPC (682.27 ms SD = 159.02) condition. No other significant differences were observed.

The participants' percentages of right responses as a function of site (PPC, Vertex), Task (A, B), and TMS (on, off) are reported in Table 1.

**Table 1:** **Percentages of right choices as a function of site (PPC, Vertex), Task (A, B) and TMS (off, on) for symmetrically (sym) and asymmetrically (asym) bisected lines**.

	**PPC**				**Vertex**			
	**Task A**		**Task B**		**Task A**		**Task B**	
	**TMS off (%)**	**TMS on (%)**	**TMS off (%)**	**TMS on (%)**	**TMS off (%)**	**TMS on (%)**	**TMS off (%)**	**TMS on (%)**
sym	28	56	50	22	28	22	78	39
asym	39	44	42	50	50	50	44	47

Overall participants showed high accuracy (91.5%) on the asymmetric stimuli, with no significant differences across conditions. The most interesting result relates to subjects' performance on the symmetrically bisected lines. For the PPC condition, the participants' choices were consistent across the two opposite tasks. In Task A during active TMS, they tended to more often choose the right segment as longer (10/18) with respect to TMS off trials (5/18) and, consistently, in Task B during active TMS, they tended to more often choose the left segment as shorter (14/18) with respect to TMS off trials (9/18). In other words, within each task instruction, the participants showed a (non-significant) tendency to underestimate the left segment during active TMS with respect to baseline trials. For the vertex condition, participants' responses were not consistent across tasks during stimulation, while they were consistent for baseline TMS off trials, during which they showed a relative leftward bias (pseudoneglect), as expected in healthy participants (Jewell and McCourt, 2000). In Task A during active TMS, they more often judged the left segment as longer than the right segment (14/18) showing the same behavior (leftward bias, i.e., pseudoneglect) of baseline off trials (13/18). On the other hand, in Task B during active TMS, they judged the same left-sided segment as shorter (11/18) leading to a significantly (*p* = 0.041) different behavior with respect to baseline trials, in which, consistently with task A, the left segment was judged as shorter less often (4/18). In other words, when TMS was active over the vertex, the participants tended to more often choose the left segment than the right segment under both task instructions. According to Bisiach et al. (1998) such a behavior would be due to a response bias, i.e., a bias in reporting preferentially one side of space independently of the specific task requests.

In order to analyse the overall participants' performance, shorter task responses were converted into longer task responses and data from the two opposite conditions were pooled together. Overall, for the PPC condition, the proportion of times the right segment was judged as longer than the left segment was significantly (*p* = 0.033) higher during active TMS (67%) with respect to off trials (39%). Thus, during PPC TMS, the participants showed a rightward bias (i.e., left segment underestimation) which was qualitatively similar to the perceptual bias shown on this task by patients with left neglect. For the vertex condition, the proportion of times the right segment was judged as longer than the left segment did not significantly differ between TMS on (42%) and off (25%) conditions.

The absence of a significant rightward bias by PPC TMS on the asymmetric lines is consistent with previous findings (Ellison et al., 2004) and may likely be explained by a ceiling effect (the test was too easy to uncover subtle TMS effects).

**Figure 2:**
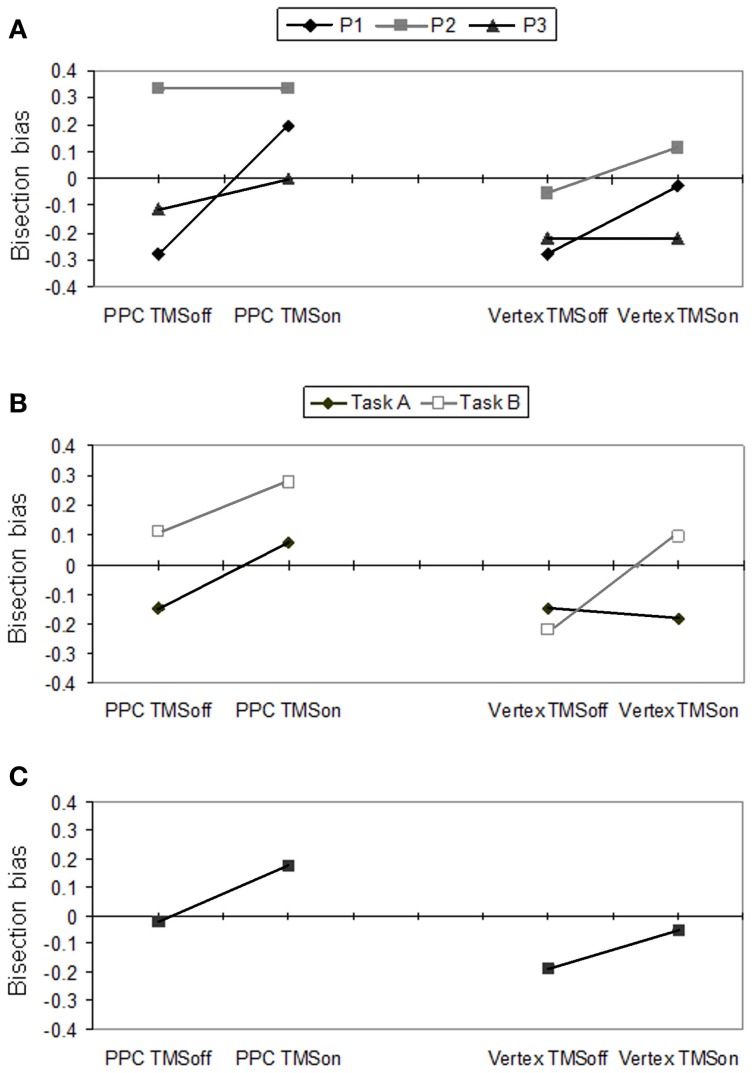
**Behavioral effects according to Fierro et al.'s (2001) score.** Right PPC and vertex stimulation for on vs. off trials. **(A)** Individual scores for P1, P2 and P3, averaged across tasks A and B. **(B)** Mean participants' scores for task A and B. **(C)** Mean participants' scores pooled across tasks.

The participants' performance as scored through Fierro et al.'s method (2001), is reported in Figure 2.

In Figure 2A, individual performances (averaged across tasks) are reported. Single cases analysis showed that P1 had a significant (*p* = 0.022) perceptual rightward bias during PPC TMS (+0.194, SD = 0.622) with respect to off trials (−0.2778 SD = 0.521). For this condition, she showed a rightward bias by PPC TMS in both tasks but this bias was significant (*p* = 0.008) only for task A (TMS on = +0.111 SD = 0.900; TMS off = −0.333 SD = 0.970). Overall, for the vertex stimulation, she did not show any significant difference between TMS on and off trials. However, vertex TMS induced a significant (*p* = 0.008) rightward bias in task B (TMS on = +0.222 SD = 0.548; TMS off = −0.222 SD = 0.548), while it did not affect the leftward bias P1 showed in task A. P1's behavior across tasks during vertex TMS is compatible with the induction of a leftward response bias. Participants 2 and 3 did not show significant differences between conditions. However, by looking at their performances averaged across tasks (Figure 2A), P3 showed a tendency toward a rightward bias during PPC TMS, and no bias in vertex TMS, while P2 showed the opposite trends.

As a group, participants showed significant differences between PPC and vertex stimulation conditions (*p* = 0.007), and between task A and B (*p* = 0.045) according to the Sign test. The difference between TMS on and off conditions did not reach a significant level. Overall, the group of participants showed a rightward bias for the PPC (mean = +0.074; SD = 0.431) with respect to the vertex condition (−0.120; SD = 0.385) and for task B (+0.056; SD = 0.397) with respect to task A (−0.102; SD = 0.433).

For PPC stimulation, there was a significant difference between TMS on and TMS off (*p* = 0.04) in the expected direction: participants produced a rightward bias (underevaluation of the left side of the line) during parietal TMS (+0.176; SD = 0.592) with respect to TMS off trials (−0.019; SD = 0.532). Also the rightward bias was near significant (*p* = 0.052) for task A.

For the vertex stimulation the difference between TMS on and off conditions was not significant. However, for task B, TMS on and off conditions were significantly different (*p* = 0.011), showing a rightward bias during active TMS (+0.074; SD = 0.578) with respect to off trials (−0.222; SD = 0.604). In task A there was a non-significant tendency in the opposite direction (TMS off = −0.148 vs. TMS on = −0.185). The most plausible interpretation of the behavioral inconsistency between performances under the two complementary tasks is that vertex TMS induced a response bias in reporting preferentially the left side. Indeed, a preference in choosing the left segment as the longer segment (as in task A) leads to leftward bias, while a preference in choosing the left segment as the shorter segment (as in task B) leads to rightward bias.

### fMRI

#### Single cases analyses

**Figure 3:**
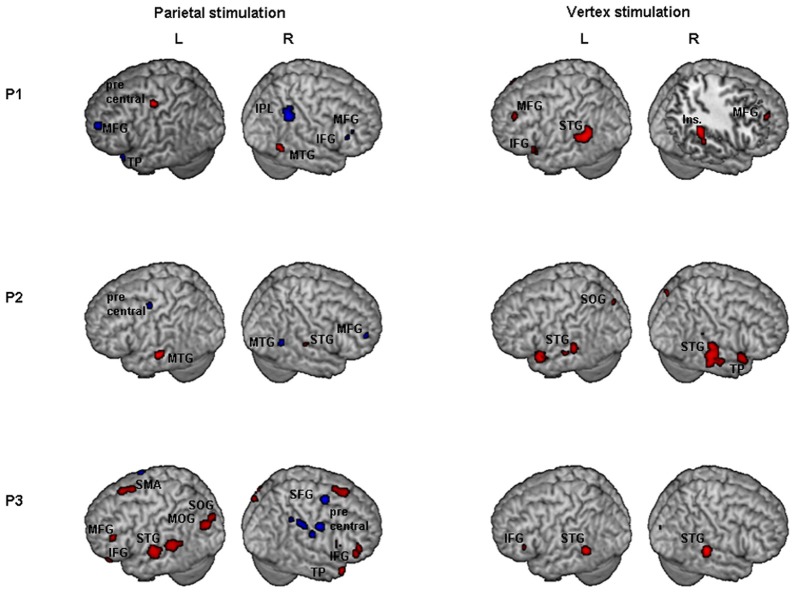
**Individual fMRI results.** Neural effect of task execution with vs. without TMS for the parietal and vertex stimulation: Participant 1, P1; Participant 2, P2; Participant 3, P3. Blue and red indicate areas with a significantly (*P* < 0.001 uncorrected; cluster level *P* < 0.05 corrected) reduced or increased neural activity, respectively. AG, Angular Gyrus; IFG, inferior frontal gyrus; Ins, insula; IPL, inferior parietal lobule; MFG, middle frontal gyrus; MOG, middle occipital gyrus; MTG, middle temporal gyrus; SFG, superior frontal gyrus; SMA, supplementary motor area; SMG, supramarginal gyrus; SOG, superior occipital gyrus; STG, superior temporal gyrus; TP, temporal pole.

Results of comparisons of BOLD signal changes during task execution with vs. without TMS separately for PPC and vertex TMS for P1, P2, and P3 are reported in Figure 3 and Table 2.

**Table 2:** **Individual comparisons of task execution with vs. without transcranial magnetic stimulation (TMS) for parietal (PPC) and vertex stimulation for Participants 1, 2, and 3 (P1, P2, and P3 respectively)**.

**Region of activation**	Coordinates^*^			***Z*-scores**
**P1: PPC—TMS ON vs. OFF**				
R middle temporal gyrus	62	−58	4	4.02
L precentral gyrus	−34	−12	54	3.82
**P1: PPC—TMS OFF vs. ON**				
R inferior parietal lobule	52	−46	46	5.25
L middle frontal gyrus	−30	58	16	4.21
L precuneus	−8	−64	46	4.12
L temporal pole	−34	24	−28	4.11
R middle frontal gyrus	56	36	24	3.60
R inferior frontal gyrus	62	28	18	3.51
**P1: VERTEX—TMS ON vs. OFF**				
L superior temporal gyrus	−54	−46	16	4.52
R insula	46	−40	20	4.38
R middle frontal gyrus	40	46	36	3.85
L temporal lobe/sub-gyral	−34	−38	6	3.58
L inferior frontal gyrus	−54	20	−4	3.57
L middle frontal gyrus	−28	44	30	3.37
**P1: VERTEX—TMS OFF vs. ON**				
R anterior cingulate gyrus	16	48	20	3.60
**P2: PPC—TMS ON vs. OFF**				
L middle temporal gyrus	−64	−20	−2	3.94
R superior temporal gyrus	54	−24	10	3.56
R temporal lobe/sub-gyral	42	−10	−16	3.55
**P2: PPC—TMS OFF vs. ON**				
R rectus gyrus	16	24	−12	4.01
L superior occipital gyrus	−24	−66	26	3.92
R middle frontal gyrus	30	50	4	3.80
L precentral gyrus	−30	−10	54	3.79
L orbitofrontal cortex	−12	52	−20	3.75
R middle temporal gyrus	56	−56	12	3.63
**P2: Vertex—TMS ON vs. OFF**				
L superior temporal gyrus	−42	−8	−10	4.96
R superior temporal gyrus	68	−26	2	4.48
L inferior frontal gyrus	−18	12	−26	4.35
L middle temporal gyrus	−62	−32	10	3.93
R temporal pole	58	14	−10	3.73
R orbitofrontal cortex	42	28	−10	3.71
R precuneus	0	−60	56	3.68
R insula	38	8	−8	3.62
L superior occipital gyrus	−10	−82	44	3.60
L precuneus	−16	−52	54	3.55
L cuneus	−6	−86	18	3.55
R cuneus	14	−88	32	3.47
**P3: PPC—TMS ON vs. OFF (EXTENDED CLUSTERS 30 VOXELS)**				
L helschl's gyrus	−40	−20	10	5.49
L inferior frontal gyrus/orbitofrontal cortex	−48	46	−18	5.06
R inferior frontal gyrus/orbitofrontal cortex	40	42	−2	4.84
R inferior frontal gyrus/frontal operculum	46	18	10	4.49
R temporal pole	38	22	−40	4.48
L superior temporal gyrus	−62	−38	18	4.47
L middle occipital gyrus	−40	−84	34	4.30
L rolandic operculum	−40	4	14	4.23
R anterior cingulate	6	2	−4	4.17
R superior temporal gyrus	36	0	−18	4.14
L supplementary motor area	2	26	58	4.06
R insula	46	−6	−2	3.90
L superior occipital gyrus	−20	−88	32	3.70
L lingual gyrus	−10	−40	−4	3.32
**P3: PPC—TMS OFF vs. ON**				
R superior frontal gyrus	28	0	66	4.36
R precentral gyrus	46	−6	36	4.28
R supramarginal gyrus	54	−14	28	3.81
**P3: Vertex—TMS ON vs. OFF**				
R hippocampus	18	−32	10	4.21
R superior temporal gyrus	60	−32	8	4.21
L hippocampus	−26	−36	12	4.20
L middle temporal gyrus	−64	−48	10	3.82
L superior temporal gyrus	−40	−36	14	3.77
R lingual gyrus	18	−76	4	3.75
L inferior frontal gyrus	−42	30	4	3.71
L frontal lobe/sub-gyral	−22	−42	36	3.68
R calcarine cortex	16	−88	12	3.62
L fusiform gyrus	−34	−14	−22	3.48
L temporal lobe/sub-gyral	−28	−66	18	3.48
L calcarine cortex	−4	−84	2	3.35
**VERTEX—TMS OFF vs. ON**				
L brainstem	0	−24	−6	3.41

TMS, transcranial magnetic stimulation; PPC, parietal; P1, Participant 1; P2, Participant 2, P3, Participant 3; R, Right; L, Left.

* Peak activity coordinates are given in MNI space; extended clusters 15 voxels, p < 0.001, uncorrected; cluster level p < 0.05 corrected.

For the PPC condition, P1 showed significant TMS-induced BOLD signal decreases in right IPL, MFG, and IFG. Decreased BOLD signal was also found in left MFG, SFG, precuneus, and superior temporal pole. Increased BOLD responses were only found in right MTG and left precentral gyrus. At the behavioral level P1 was the only participant who showed a significant rightward bias during right PPC TMS.

During parietal TMS a similar pattern of right parieto-frontal deactivations (SMG, SFG, and precentral gyrus) was found for P3, who at behavioral level showed a non-significant tendency toward a rightward bias. In this condition P3 showed increased BOLD signal in many regions (for details see Table 2).

P2 did not show any decreased BOLD signal in right PPC during parietal TMS. However, as P1, P2 showed decreased BOLD activity in right MFG. Interestingly at a behavioral level this participant did not show any significant rightward bias or tendency toward it during PPC TMS.

To summarize, during PPC TMS, single case analyses showed right parieto-frontal decreased activity for the two participants whose PPC site was found to overlay the right AG (P1 and P3) and that at a behavioral level, manifested a significant rightward bias (P1) or a non-significant tendency toward it (P3). In contrast, neuroimaging results for P2, whose parietal site was found to target a slightly higher PPC spot, overlying the SPL near the IPS, and whose performance was not biased, did not show any decreased activity in PPC. All three participants had decreased activity in the right frontal cortex. Single case behavioral and neuroimaging findings suggest a crucial role within the right PPC of IPL sites and in particular of the AG in the causation of orientation biases.

Concerning vertex stimulation, all three participants showed patterns of bilateral increased BOLD signal in cortical regions comprising the temporal, frontal, and occipital cortex, sparing the IPL. The locus of decreased BOLD signal during vertex stimulation was limited to a single site for P1 and P3 (no significant result at this threshold was found for P2, for details see Table 2).

P1 was the only participant who showed a significant behavioral bias during vertex stimulation in task B, which could be explained by a leftward response bias. The group analyses (see below) showed in this condition an increased activity in the left caudate, a site which has been linked to response bias in neglect patients (Vossel et al., 2010). Even though for P1 at the statistical threshold used for single cases analysis, increased activity was only found in cortical regions, subsequent analyses using a lower threshold (*p* = 0.01 uncorrected, extended cluster size = 20) showed significant activation in the left caudate (MNI: −20, 12, 22).

#### Group analysis

**Figure 4:**
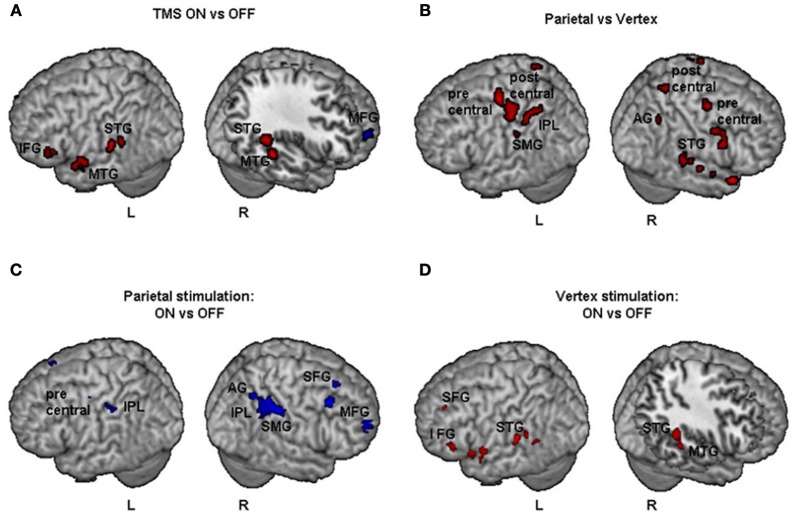
**Group fMRI results during task execution for (A) TMS on vs. TMS off (independently of the site of stimulation), (B) parietal vs. vertex stimulation (independently of on off trials), (C) parietal: TMS on vs. TMS off, (D) vertex: TMS on vs. TMS off.** Blue and red indicate areas with a significantly (*P* < 0.001 uncorrected; cluster level *P* < 0.05 corrected) reduced or increased neural activity, respectively. AG, Angular Gyrus; IFG, inferior frontal gyrus; IPL, inferior parietal lobule; MFG, middle frontal gyrus; MTG, middle temporal gyrus; SFG, superior frontal gyrus; SMG, supramarginal gyrus; STG, superior temporal gyrus.

Results of comparisons of BOLD signal changes for TMS on vs. off are reported in Figure 4A. During TMS higher activations of bilateral superior and middle temporal gyri (STG and MTG respectively) and of left inferior frontal gyrus (IFG), hippocampus, and cerebellum were found. Decreased neural activity was found in the right MFG and left anterior cingulate cortex (ACC). Bilateral increased activation of temporal cortex during stimulation might be explained by peripheral effects due to the sound of TMS.

The comparisons of activations during PPC condition with respect to vertex condition (independently of on/off trials) brought about a widespread pattern of right hemisphere activations together with more circumscribed activations of the left hemisphere (Figure 4B). In particular, higher activation during PPC with respect to vertex condition was found in the right AG, left inferior parietal lobule (IPL) and supramarginal gyrus (SMG). These results are consistent with the idea that the effects of brain stimulation also depend on the ongoing state of the stimulated cortex, i.e., how excitable the cortex is at the time of stimulation (Siebner et al., 2009). Indeed, right PPC is specifically involved in line bisection judgments (Fink et al., 2000) while this is not the case for the vertex site.

Comparisons between tasks (A vs. B) did not give any significant result at the threshold used for the other contrasts.

Results of comparisons of BOLD signal changes during task execution with vs. without TMS separately for PPC and vertex stimulation are reported in Table 3 and Figures 4C and D, respectively.

**Table 3:** **Group analysis: comparisons of task execution with vs. without transcranial magnetic stimulation (TMS) for parietal (PPC) stimulation and vertex stimulation**.

**Region of activation**	Coordinates^*^			***Z*-scores**
	***X***	***Y***	***Z***	
**PPC—TMS ON vs. OFF**				
L cerebellum	−22	−46	−16	5.73
L rolandic operculum	−42	4	16	4.90
L cingulate gyrus	−4	4	28	4.88
**PPC— TMS OFF vs. ON**				
R supramarginal gyrus	54	−32	46	4.67
R middle frontal gyrus	34	20	40	4.65
R supplementary motor area	10	−18	56	4.52
R inferior parietal lobule	44	−42	48	4.38
R frontal lobe/sub-gyral	28	−38	30	4.62
L frontal lobe/sub-gyral	−22	−8	38	4.27
R angular gyrus	30	−56	46	4.10
L precentral gyrus	−22	−22	54	4.08
R superior frontal gyrus	20	28	54	3.75
L inferior parietal lobule	−48	−34	44	3.62
**VERTEX—TMS ON vs. OFF**				
R middle temporal gyrus	52	−38	4	4.42
R superior temporal gyrus	48	−42	14	3.72
L superior frontal gyrus	−18	42	28	4.20
L superior temporal gyrus	−56	−30	12	4.10
L insula	−44	14	−10	3.51
L inferior frontal gyrus	−42	32	−4	3.96
L caudate	−18	−24	18	3.92
L parietal lobe/sub-gyral	−36	−38	24	3.91
L temporal lobe/sub-gyral	−40	−40	−10	3.89
L hippocampus	−18	−4	−12	3.81
**VERTEX— TMS OFF vs. ON**				
R anterior cingulate gyrus	6	50	24	4.18

TMS, transcranial magnetic stimulation; R, Right; L, Left.

* Peak activity coordinates are given in MNI space; extended clusters 18 voxels, p < 0.001, uncorrected; cluster level p < 0.05 corrected.

For the PPC condition during task execution (Figure 4C and Table 3), significant TMS-induced BOLD signal decrease was found in the right AG, SMG, IPL, supplementary motor area (SMA), MFG, and superior frontal gyrus (SFG). Decreased BOLD signal was also found in the left IPL and precentral gyrus. Only a few areas of increased BOLD response were found in the left rolandic operculum, cingulate cortex, and cerebellum. Since at behavioral level, the difference between on and off trials during task execution was significant for task A, a comparison of TMS on and off conditions was also performed for this task. The results showed significant (*Z* > 3.60) decreased BOLD signal in right SMG (MNI coordinates: 54, −32, 46), IPL (44, −42, 48), SMA (14, −16, 56), MFG (30, 66, 14), and middle occipital gyrus (MOG, 34, −84, 12).

For the vertex condition (Figure 4D and Table 3), during task execution at same threshold, we found a decreased TMS-induced BOLD signal only in the right ACC. By contrast, there were several areas of increased BOLD response in the bilateral temporal cortex and left hemisphere cortical (SFG, IFG, insula, and hippocampus) and subcortical regions (caudate). Since at behavioral level there was a significant bias in task B, a comparison of on vs. off conditions for this task was performed. Results showed increased TMS-induced BOLD signal (*Z* > 3.28) in left inferior temporal gyrus (ITG, −38, −38, −12), left caudate (−18, −24, 22), left hippocampus (−32, −36, 0), and sub-gyral temporal cortex (−36, −46, 0).

## Discussion

The results of the present study demonstrate that the method proposed here is feasible and could provide insight for future TMS/fMRI studies aimed at tracing *in vivo* TMS effects on brain activation underlying behavioral changes.

Although future investigations are necessary to validate and further explore these preliminary findings, individual and group analyses show for the first time the involvement of a fronto-parietal network in the induction of neglect-like behavior on the landmark task by TMS over PPC region. Even though these results are clearly preliminary, given the extremely low number of participants, they provide converging evidence with recent findings supporting new functional-network accounts (Ruff et al., 2009b; Driver et al., 2010) of spatial attention and neglect (Bartolomeo, 2006; Bartolomeo et al., 2007; Doricchi et al., 2008).

In accordance with previous data (Brighina et al., 2002), right PPC TMS affected participants' performance at “perceptual” rather than “response” level of spatial processing. The observation of right parieto-frontal decreased activity during TMS perceptual bias is in line with TMS (Brighina et al., 2002) and stroke patient findings suggesting that both parietal and frontal regions are implicated in sensory neglect, while basal ganglia would be implicated in directional hypokinesia (Bisiach et al., 1998; Sapir et al., 2007; Vossel et al., 2010). Changes in brain activity comprised areas that have been found to underlie line bisection judgments on the landmark task in healthy participants (Fink et al., 2000).

Overall the participants did not show any significant bias during vertex stimulation when performances from the two opposite tasks were collapsed. However, in contrast to what occurred during parietal TMS, during vertex TMS, performances were inconsistent across tasks. Indeed, participants reported more often the left segment as shorter in task B (producing a rightward bias) and the same (left-sided) segment as longer in task A (producing a leftward bias not different from baseline performance). Participants' performances were consistent across tasks during baseline conditions (without TMS), on which they showed the leftward bias (pseudoneglect) typically produced by healthy subjects (Jewell and McCourt, 2000). According to findings by Bisiach et al. (1998), the most parsimonious interpretation of these results is that vertex TMS induced a leftward response bias, i.e., a bias in reporting preferentially the left segment, independently of the specific task requests. Neuroimaging analyses for this condition showed a main pattern of BOLD signal increases, comprising bilateral temporal cortex and left hemisphere cortical and subcortical regions. In particular, increased BOLD signal was found in the left caudate. Right caudate lesions have been recently linked to rightward response bias on the landmark task in neglect patients (Vossel et al., 2010). In the present study, vertex stimulation might have induced a leftward response bias by interfering with the left caudate (right hand response) ongoing activity. Reduced BOLD signal was only found in the right ACC. This outcome could be due to ACC functional connectivity with cortical regions stimulated by vertex TMS and its involvement in spatial attention RTs tasks execution. It is possible that the reduced neural activity in ACC during stimulation and cumulative after-effects by single pulses during trials without stimulation, might in part explain the slower RTs which were found for the overall vertex condition with respect to the PPC condition (independently of delivery of TMS pulses and for both symmetrically and asymmetrically bisected lines).

The network extended pattern of reduced BOLD activity observed during PPC TMS and rightward bias on the Landmark task is consistent with current models of spatial attention in humans suggesting the importance of fronto-parietal network for spatial attention in the right hemisphere (Corbetta and Shulman, 2002; Kincade et al., 2005; Chica et al., 2011). Indeed, evidence that attentional processes are involved in horizontal length estimations has been provided in neglect patients (Urbanski and Bartolomeo, 2008) and in healthy participants (Toba et al., 2011). According to the above models endogenous and exogenous spatial orienting operate through the activity of a dorsal bilateral fronto-parietal network. A more ventral right attentional network is involved in responding, along with the dorsal network, to behaviorally relevant objects and reorienting. Core regions of the dorsal network are the IPS, the SPL and the dorsal frontal cortex along the precentral sulcus, near or at the human frontal eye fields. The ventral network comprises the TPJ and ventral frontal cortex. An area that would belong to both systems is the MFG. The anatomical projections of the human homologue of the dorsal superior longitudinal fasciculus (SLF I) and (ventral) SLF III would overlap with the dorsal and the ventral attentional networks respectively, while (middle) SLF II would comprise the parietal component of the ventral network and the prefrontal component of the dorsal network (Thiebaut De Schotten et al., 2011).

In the present study, decreased neural activity during right PPC TMS comprises regions belonging to both dorsal and ventral attentional networks. A possible interpretation of these findings is that decreased activity during right PPC TMS overlapped with parieto-frontal regions which are directly linked through the likely human homologue of SLF II (Thiebaut De Schotten et al., 2011). Indeed, right hemisphere decreased BOLD signal comprised the AG (a structure corresponding to the monkey caudal inferior parietal lobe where SLF II has been shown to originate), parietal components of the ventral network (SMG) and frontal components of the dorsal network (MFG and SFG). The interpretation that brain deactivations during right PPC TMS and rightward bias involved parietal and frontal components of SLF II would be consistent with the hypothesis that this tract plays a crucial role in attentional processes underlying line bisection performance. Intraoperative electrical stimulation of a subcortical site corresponding to right SLF II has been shown to induce dramatic rightward line bisection shifts in patients during brain surgery (Thiebaut De Schotten et al., 2005; Bartolomeo et al., 2007). In addition, right hemisphere SLF II volumes have been found to positively correlate with leftward bisection biases (i.e., pseudoneglect) in healthy participants (Thiebaut De Schotten et al., 2011).

Right PPC TMS also induced changes in the contralateral hemisphere. Reduced activity was found in the IPL and precentral frontal cortex, while increased activity involved the cerebellum, the rolandic operculum and the cingulate gyrus. Although the observation of increased activity in contralateral regions during suppression of ipsilateral cortex is consistent with the rivalry account of inter-hemispheric interactions (Kinsbourne, 1977), the finding of reduced activity in left parieto-frontal areas is in contrast with this. However, this outcome is consistent with recent TMS findings suggesting the existence of other forms of inter-hemispheric interactions (Blankenburg et al., 2008), in line with the physiology of the corpus callosum characterized by a predominance of excitatory inter-hemispheric connections. For instance, some studies show that prolonged low frequency rTMS induce bilateral decreases of cortical excitability (Wassermann et al., 1998; Nowak et al., 2008).

In this study, right PPC TMS might have induced first neuron synchronization followed by long lasting inhibition at the site of stimulation (Moliadze et al., 2003). As a consequence, the impact of TMS on the site of stimulation may have lowered activity in its efferent intra- and inter-hemispheric projections to anatomically connected regions. Decreased activity in right parietal and frontal regions might have led to release from inhibition other contralateral regions as predicted by the rivalry account of inter-hemispheric influences.

Results of this preliminary study are consistent with the literature on neglect neuroanatomy. During right PPC TMS and induction of rightward bias reduced neural activity was observed in posterior parietal and frontal areas whose lesions or altered functionality have been implicated in neglect symptomatology by localization studies (Heilman and Valenstein, 1972; Vallar and Perani, 1986; Husain and Kennard, 1996; Mesulam, 1999; Mort et al., 2003; Karnath et al., 2004; Hillis, 2006). In particular, these findings suggest the importance of right inferior parietal sites in the causation of rightward orientation bias, as often pointed out by the neglect literature (Leibovitch et al., 1998; Vallar, 2001; Maguire and Ogden, 2002; Marshall et al., 2002; Mort et al., 2003; Medina et al., 2009; Verdon et al., 2010). They also seem to support the evidence that neglect symptoms are accounted for by functional breakdown of connectivity within attentional networks (He et al., 2007) and that damage to SLF, which disconnects parietal and frontal cortices, is significantly involved in the causation and severity of the neglect syndrome (Doricchi and Tomaiuolo, 2003; Bartolomeo et al., 2007; Doricchi et al., 2008).

The observation of bilateral IPL deactivation in concomitance of TMS-induced neglect-like bias might suggest that the pattern of hemispheric imbalance with relative hyper-activation of the unaffected and relative deactivation of the affected hemisphere, which has been hypothesized (Kinsbourne, 1977) and observed in left neglect patients (Corbetta et al., 2005), might occur over time as adaptive/maladaptive neuroplastic response to a brain lesion and exacerbate symptoms, rather than being crucial to neglect emergence. Indeed, amelioration of neglect symptoms by inhibitory rTMS over the unaffected PPC has been reported in sub-acute and chronic patients (Brighina et al., 2003; Shindo et al., 2006; Song et al., 2009). The network extended effects of online TMS might be better likened to brain changes occurring during diaschisis than neuroplastic changes following the acute phase.

The main limitations of this study are the small sample size and its composition. Indeed, participants were all females, and therefore a sample in which hemispheric asymmetries might have been less pronounced than in males (Catani et al., 2007). Both limitations worked against the emergence of behavioral and neuroimaging significant effects. Additionally, such a small sample size increases the risk that group results were largely affected by individual findings.

These limitations were due to practical and technical factors common to the design and execution of TMS/fMRI studies. Technologically more sophisticated experiments are more demanding than basic experiments. Indeed, some potential participants were excluded from the study because of decline of their performance when practicing the task inside the scanner. Another variable, which limited testing of some available participants, was their high Motor Threshold at the scanner TMS machine, which precluded the possibility to use even higher intensities. In addition, the TMS coil placement over the PPC site was much more difficult to achieve due to the restricted space within the MRI coil compared to the positioning of the coil over the vertex. In relation to this, the participant's head size was also important.

Another major limitation of the present study is the use of anatomical skull landmarks to identify the PPC site. Even though this represents an economical and practical method frequently used in TMS studies, given inter-individual differences in brain neuroanatomy and the small distance between contiguous regions around IPS, the use of this method for PPC coil placement can easily lead to target functionally distinct areas (Herwig et al., 2003). Indeed, even though the coil was accurately located at the intended site in all participants, individual *post-hoc* projections showed that for two participants the site of stimulation overlaid the AG, while in one participant it was found to target a slightly higher cortical site in the SPL near the IPS.

Large sample size TMS/fMRI investigations using hunting procedure (see for example, Bjoertomt et al., 2002) or neuronavigation systems to precisely localize a specific PPC hotspot will be necessary to overcome the limitations of this initial study.

## Conclusions

To our knowledge, this is the first time the neural correlate of single-pulse TMS inducing a behavioral bias mimicking a neuropsychological deficit has been investigated using fMRI.

Further enhancements of the TMS/fMRI technique might allow one to image disruption of neural networks that occur during different forms of behavioral bias and ultimately help design more effective treatments to restore deficits following stroke.

### Conflict of interest statement

The authors declare that the research was conducted in the absence of any commercial or financial relationships that could be construed as a potential conflict of interest.
